# Analysis and Identification of the Mechanism of Damage and Fracture of High-Filled Wood Fiber/Recycled High-Density Polyethylene Composites

**DOI:** 10.3390/polym11010170

**Published:** 2019-01-18

**Authors:** Yong Guo, Shiliu Zhu, Yuxia Chen, Dagang Li

**Affiliations:** 1 School of Forestry and Landscape Architecture, Anhui Agricultural University, Hefei 230036, China; zhuslwood@163.com (S.Z.); sheherose@163.com (Y.C.); 2 College of Materials Science and Engineering, Nanjing Forestry University, Nanjing 210037, China

**Keywords:** particle-reinforced composites, acoustic emission, damage mechanics, fracture, recycling

## Abstract

The damage and fracture of fiber reinforced polymer composites are vital constraints in their applications. To understand the mechanism of damage of wood fiber (WF) reinforced high density polyethylene (HDPE) composites, we used waste WF and recycled HDPE (Re-HDPE) as the raw materials and prepared high-filled WF/Re-HDPE composites via extrusion. The damage and fracture mode and failure mechanism of the composites with different WF contents (50%, 60%, and 70%) was studied under a three-point bending test by combining the acoustic emission (AE) technique and scanning electron microscope (SEM) analysis. The results show that AE technology can better assist in understanding the progress of damage and fracture process of WF/Re-HDPE composites, and determine the damage degree, damage accumulation, and damage mode. The damage and fracture process of the composites presents three main stages: the appearance of initial damage, damage accumulation, and destructive damage to fracture. The matrix deformation, fiber breakage, interface delamination, fiber-matrix debonding, fiber pull-out, and matrix cracking were the dominant modes for the damage of high-filled WF/Re-HDPE composites under bending load, and the AE signal changed in different damage stages and damage modes. In addition, the WF content and repeated loading had a significant influence on the composite’s damage and fracture. The 50% and 60% WF/Re-HDPE composites produced irreversible damage when repeated load exceeded 75% of the maximum load, while 25% of the maximum load could cause irreversible damage for 70% WF/Re-HDPE composites. The damage was accumulated owing to repeated loading and the mechanical properties of the composites were seriously affected.

## 1. Introduction

Natural fiber composites depend on their inherent environmental and performance advantages and have become an alternative for replacing environmentally harmful synthetic materials, and hence, help control pollution problems [1]. Especially wood fiber (WF) reinforced high density polyethylene (HDPE) composites have been widely used in arenas such as construction, automotive, garden, interior decoration, and daily life [2,3]. However, in order to employ the wood textures and reduce the cost, the WF content of the composite needs to exceed 50% in the practical production, which causes a poor interface compatibility between the WF and HDPE. The poor interface results in micro cracks inside the WF/HDPE composites and expands, causing larger cracks or even breakage under sustained loading, resulting in material scrapping or life reduction.

Studies have shown that the WF/HDPE damage and fracture behavior are closely related to the fiber/matrix interface [4]. The damage and failure modes of fiber reinforced polymer composites are mainly divided into fiber pull-out, fiber breakage and delamination, matrix cracking, and fiber-matrix debonding [5,6,7,8,9]. However, it has been difficult to accurately and effectively distinguish the failure modes and understand the evolution of the damage mechanism of high-filled WF/HDPE by using the traditional evaluation methods and testing techniques. However, the damage process would produce an acoustic emission (AE) characteristic signal from occurrence to break. AE is one of the most reliable and well-established novel techniques in non-destructive testing, which can monitor and identify the stress wave signals of the composite from the microscopic deformation stage until the fracture process. Furthermore, the accumulation of damage during the deformation and failure process is monitored, the failure mechanism is identified, and the damage location and fracture modes are determined [10,11,12,13,14].

AE technique is often used to dynamically monitor the damage and failure process of fiber reinforced polymer composites and evaluate its properties (including fracture and mechanical properties), strength, and lifespan [5,8,9,15,16,17,18]. For instance, Dogossy and Czigany [19] combined the AE and scanning electron microscope (SEM) and analyzed the failure process of polyethylene (PE) composites filled with maize hull. The relationship between the amplitude and time suggested that there were three kinds of failure modes, namely matrix deformation (below 25 dB), maize hull pull-out (26–40 dB), and maize hull breakage (over 41 dB). In another study, the authors found similar results in delaminated glass fiber epoxy composites by combining the AE technology with digital image correlation [20]. However, four micromechanical deformation processes were determined in polypropylene/wood flour composites via AE and volume strain measurements analysis. The results conclude that the matrix polymer deforms mainly by shear yielding, fibers debonding initiated at extremely small deformations and stresses, which is followed by the fiber pull-out at an intermediate stress level, and the fracture of WF is the dominating deformation process [21]. Moreover, the number and amplitude range of AE signals caused by interface failure indicated that quality of the fiber-matrix interface plays a major role in the damage process of hemp fiber reinforced polypropylene composites [4]. The AE technology can also be used to evaluate the compatibility of the fiber-matrix interface in composites [22], and there is a clear correlation between the damage mechanism of fiber reinforced polymer composites and the AE parameters [23,24,25]. In addition, the AE results demonstrated the shifting of energy dissipation from matrix cracking to fiber pullout during the damage progression [11].

Although AE technology has been effectively employed in monitoring the damage process of fiber reinforced composite, there is still limited research on the damage and fracture process of high-filled WF/HDPE composites under three point bending. However, this material is often subjected to bending loads rather than tensile loads in daily application. Therefore, the authors prepared high-filled WF/HDPE composites with different fractions of WF (50%, 60%, and 70%), and combined AE technology with SEM to monitor the three-point bending loading process. The damage and failure behavior, such as damage and fracture characteristics, damage accumulation, damage location, and failure mechanism of the composites, were analyzed via parametric analysis, waveform analysis, and characteristic waveform analysis. This has crucial practical significance for enriching the non-destructive monitoring and performance evaluation methods of high-filled WF/HDPE composites.

## 2. Materials and Methods 

### 2.1. Materials

Wood fiber (WF, Italian poplar (Populus euramevicana cv.‘I-214’), particle size 40–80 mesh, the fiber aspect ratio is 4–7, and the shape is irregular) was provided by the Jiangsu Siyang Wood Powder Factory, Siyang, China. Recycled high-density polyethylene (Re-HDPE, melt flow index = 1.23 g/10 min at 190 °C, 5 kg) was obtained from the Yixing Zhangye Town Huahong Plastic Product Factory, Yixing, China. The commercial maleic anhydride grafted polyethylene (MA-g-PE, CMG9801) and stearic acid (HSt) were purchased from Shanghai Sunny Technology Co. Ltd. Shanghai, China and Shanghai Yanan Grease Chemical Co., Ltd., Shanghai, China, respectively. The MA-g-PE is a polymer interface coupling agent with strong reactivity. It helps to improve the compatibility between WF and Re-HDPE and the dispersibility of WF. Additionally, it plays a role as a bridge between compatibility and adhesion in composite systems. The HSt is a commonly used internal lubricant for wood-plastic products. It can effectively promote the plasticization of the Re-HDPE matrix and reduce the melt viscosity, improve the processing fluidity, and improve the surface finish of the product. The MA-g-PE and HSt used as the compatibilizer and lubricant in this experiment, respectively.

### 2.2. Composite Preparation

The WF was dried at 105 °C for 12 h to reach a moisture content of less than 1%. Then, WF, Re-HDPE, and additives (MA-g-PE and HSt) were mixed uniformly in a high speed mixer (DQL-100, turbine blade and cast from 304 stainless steel; the maximum volume is 100 L, Zhangjiagang Daqin Machinery Factory, Zhangjiagang, China). The homogeneous mixture was extruded through a one-step conical twin-screw extruder (model SJZ65, Shanghai Jwell Machinery Co.,Ltd., Shanghai, China) to prepare a highly-filled WF/Re-HDPE composite. The power of the main motor of the extruder was 37 KW, and the max output was 250 kg/h. The screw operated in counter-rotating mode and there were kneading blocks in the extruder head. The screw had a diameter of 65/132 mm and the aspect ratio was 25. The extrusion process parameters were engine speed = 16–18 rpm, feeding speed = 8–10 rpm, and temperature = 150–155 °C. Three levels of composites (50% WF/Re-HDPE, 60% WF/Re-HDPE, and 70% WF/Re-HDPE composites) were prepared, where the corresponding WF content was 50%, 60%, and 70% (weight percent (wt %)), and Re-HDPE content was 47%, 37%, and 27% (weight percent (wt %)), respectively. The content of MA-g-PE and HSt was 2% and 1% (weight percent (wt %)) for each level. The prepared composite sheet was processed into a specimen with the size of 500 mm (L) × 20 mm (T) × 30 mm (R) using a precision push bench saw (CS70EB, Festool, Wendlingen, Germany).

### 2.3. Characterization

**Figure 1:**
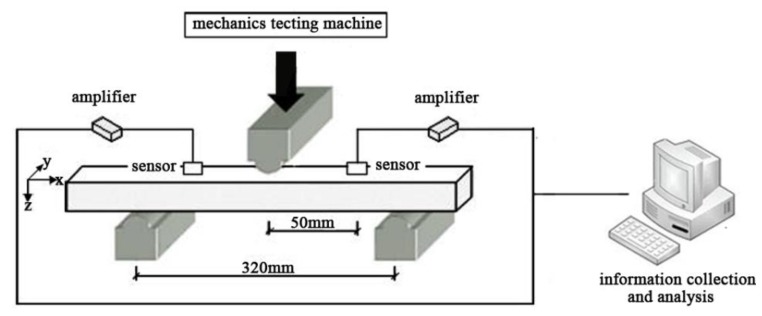
Schematic of the three-point bending test and the acoustic emission signal acquisition.

The specimen was kept in a landscape orientation and pressurized in a three-point bending mode using a universal testing machine (AUTOGRAPH AG-IC, Shimadzu, Kyoto, Japan) (shown in Figure 1). The span was 320 mm, while the loading speed was 3 mm/min. The AE signals produced by the stressed specimen were collected using the AE signal acquisition system (PCI-2, Physical Acoustics Corporation (PAC), Princeton, New Jersey, USA). The PCI-2 AE acquisition system was divided into three parts: sensor, preamplifier, and high speed acquisition card. The sensor model was R15 while the resonant frequency was 150 kHz. The sensor was fixed on the surface of the specimen (the distance between the sensor and the center of the specimen was 50 mm) and coupled with a coupling agent to ensure good contact with the specimen, and the detection threshold was 30 dB. The AE signal was not collected when the amplitude was lower than the detection threshold (30 dB), and gradually collected as the amplitude became higher than 30 dB. The preamplifier uses PAC’s 2/4/6 type to provide amplification of three gains of 20/40/60 dB, and the gain in this experiment was chosen as 40 dB. The sensor receives the AE signal, transmits it to the preamplifier, and then collects and records through the computer equipped with the AE acquisition card. The laboratory remained quiet and noise free at the test temperature of 25 °C and a relative humidity of 30–40% throughout the experiment.

For the repeated loading bending tests, the maximum load was set at 25%, 50%, and 75% of the average maximum breaking load in the three-point bending test. The loading test was repeated three times and the specimen was broken during the fourth load. To determine the damage and fracture mode of the high-filled WF/Re-HDPE composite, the fracture surface of the specimen was observed using scanning electron microscopy (SEM, S-4860, Hitachi, Tokyo, Japan). The SEM operated at an accelerating voltage of 10.0 kV and the fractured surfaces of the specimens were coated with a thin layer of gold prior to the SEM analysis.

## 3. Results and Discussion

### 3.1. AE Signal Analysis of Composites Damage and Fracture Mode

**Figure 2:**
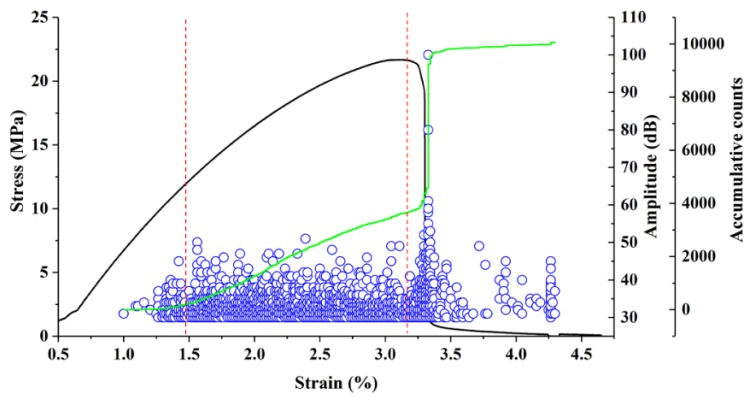
Acoustic emission (AE) accumulative counts vary with stress-strain during three-point bending test. The black curve represents stress-strain curve; the green curve represents AE accumulative counts; and the blue circle represents amplitude.

**Figure 3:**
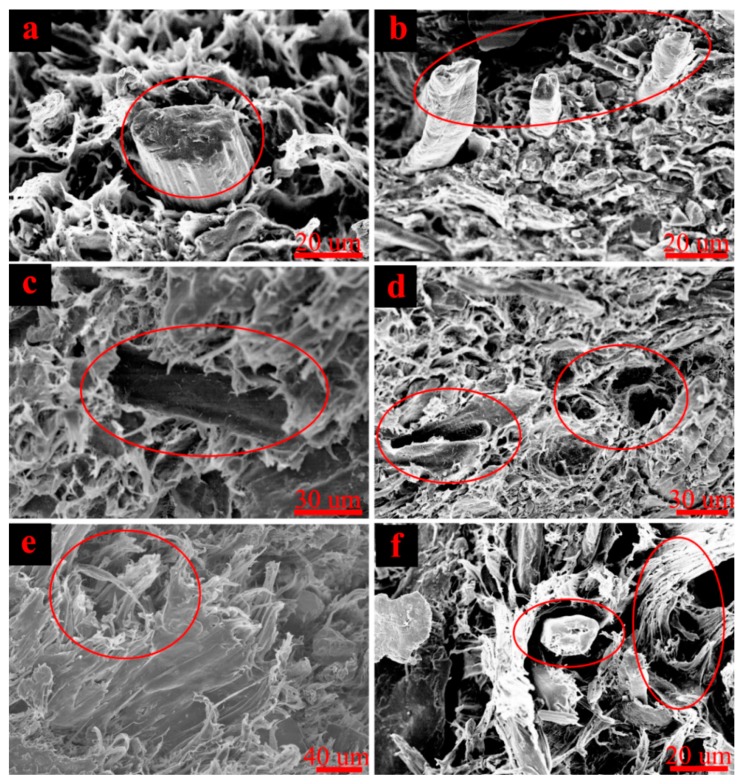
Scanning electron microscope (SEM (analysis of damage and fracture mode and characteristics ((**a**,**b**)—Fiber breakage; (**c**,**d**)—Fiber pull-out (interface friction); (**e**)—Matrix cracking; (**f**)—Fiber-matrix debonding (interface delamination) and matrix deformation).

Figure 2 shows that the AE accumulative energy varies with stress-strain during the three-point bending test. The bending procedure of high-filled WF/Re-HDPE composites consisted of three steps: line elastic deformation, nonlinearity deformation, and fracture. The line elastic deformation was short, while the nonlinearity deformation was long and showed a rapid decline when stress reached the maximum value. The SEM fracture analysis (see Figure 3) of the bending specimen shows that the damage modes were: matrix deformation, fiber breakage, interface delamination (fiber debonding), fiber pull-out, and matrix cracking. In addition, the fracture was relatively uniform and belonged to brittle failure [26,27].

The study of the variation of AE signal parameters with stress-strain in the damage stage presents four stages: (1) initial damage, where there is a little AE signal generation, and a small number of AE signals appear at the late loading, indicating that the internal damage and AE event of the composite is extremely small, the released energy is minuscule at the initial stage; (2) slow damage growth, where the AE signal begins to show significant fluctuations, indicating that the damage in the composite begins to accumulate and grow as the stress increases, and the released energy due to damage begins to increase and tends to stabilize; (3) gradual intensification of damage, where the damage inside the composite is further increased with the increasing stress, and the AE event is gradually increased, indicating that the released energy is significantly increased; (4) sharp rise in damage, where both the AE event and the strain energy released by the fracture rise sharply.

**Figure 4:**
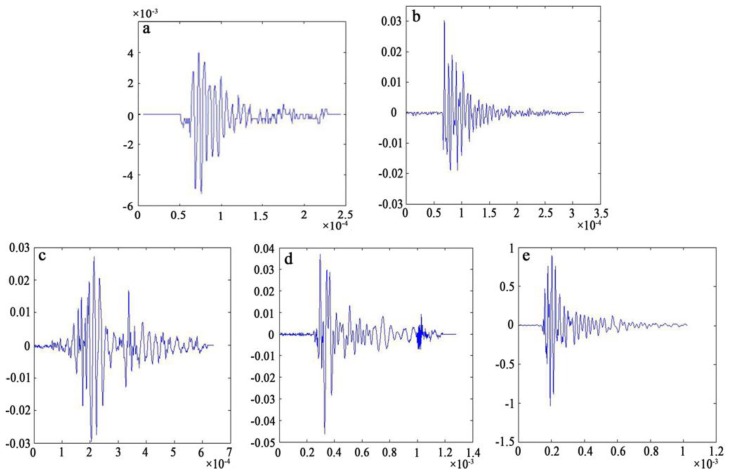
Five AE characteristic waveforms corresponding to the damage and fracture mode. (**a**) Type I (Matrix deformation); (**b**) Type II (Fiber breakage); (**c**) Type III (Interface delamination); (**d**) Type IV (Interface friction); and (**e**) Type V (Matrix cracking).

A preliminary analysis of the pre-collected AE signals of the high-filled WF/Re-HDPE composite shows that frequencies were mainly within 400 KHz. Therefore, the sampling rate was set to 2 MHz to ensure that the collected signals conformed to the Nyquist sampling theory and that the original signals were well recovered. For further study, the signal was decomposed and denoised by wavelet transform, and then reconstructed for each failure stage; the obtained five characteristic waveforms are shown in Figure 4. The five damage and fracture modes of the damage process were determined by combining the distribution of five AE characteristic waveforms and SEM fracture analysis. These damage modes had the following waveform characteristics. The initial AE signal produced in the bending test was type I, which was distributed throughout the AE process and was the dominant waveform, showing lower amplitude (general amplitude was lower than 40 dB, average amplitude was 34.31 dB) and energy. Type II began to appear sporadically in the initial nonlinearity deformation stage, gradually increased, and occurred throughout the damage and fracture process showing short rise time (generally less than 5 μs, average was 2.13 μs), high amplitude (generally above 50 dB, average was 55.29 dB), and high energy (average was 339.31 × 10^−9^ J); the average duration was 108.47 μs and ringing count (average was 13.70 times) was significantly higher than type I. Type III also began to appear in the nonlinearity deformation stage and later than type II, however, the occurrence frequency was higher than type II, especially in the later nonlinearity deformation and fracture stage, showing higher rise time (average was 40.43 μs) and high duration (average was 252.72 μs); the energy (average was 229.54 × 10^−9^ J) was slightly lower than the type II, general amplitude was higher than 40 dB, average amplitude was 45.61 dB, and ringing count (average was 24.96 times) was significantly higher than type II. Type IV primarily appeared with the interface delamination and fiber pull-out, showing high duration (average was 864.25 μs), high energy (average was 25.7.12 × 10^−9^ J), high ringing count (average was 39.53 times), high amplitude (average was 48.36 dB), and the average rise time was 75.925 μs. Type V appeared after Type III and mainly in the fracture, but less frequently, showing extremely high amplitude, energy, rise time, duration, and ringing counts, as shown in Table 1.

**Figure 5:**
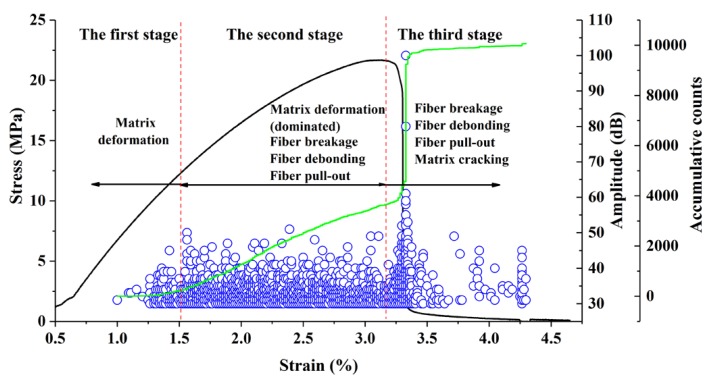
Distribution of damage and fracture stage and mode. The black curve represents stress-strain curve; the green curve represents AE accumulative counts; and the blue circle represents amplitude.

These results suggest that the damage and fracture process of WF/Re-HDPE composites under the three-point bending test can be summarized into three stages (Figure 5). In the first stage, the matrix of WF/Re-HDPE composites deformed under the bending load and varied AE signals (mostly at low amplitude and low energy) were induced by different matrix deformations. In the second stage, the matrix deformation accelerated as the loading continued. The fiber breakage and fiber debonding occurred in the meantime and cracks in the composites were generated and expanded. The Fiber debonding gradually increased in the later part of the second stage and was accompanied by fiber pull-out. The fiber breakage appeared earlier than the fiber debonding and the AE signal of fiber breakage mainly showed high amplitude and a short rise time. In the third stage, fiber debonding and fiber pull-out intensified with the increasing load which was accompanied by matrix cracking until the specimen fracture. The AE signal of interface friction generated due to fiber pull-out was mainly manifested as relatively low amplitude, whereas the matrix cracking manifested with a high amplitude. Moreover, the AE signals of interface friction and matrix cracking also showed high energy, long duration, long rise time, and large ringing counts.

### 3.2. Relationship between AE Signal and Stress-Strain of WF/Re-HDPE Composites

**Figure 6:**
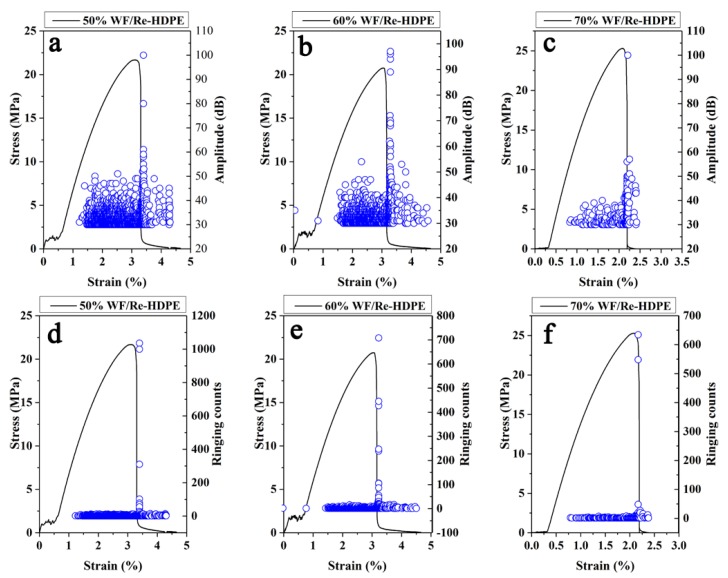
The AE amplitude, ringing counts, and stress-strain curves of wood fiber (WF)/recycled high density polyethylene (Re-HDPE) composites with different WF contents. The blue circle represents amplitude and ringing counts in (**a**–**f**), respectively.

**Figure 7:**
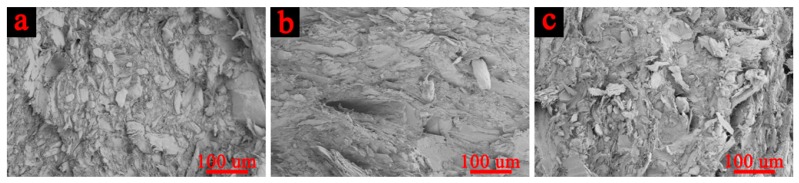
The interface morphology of WF/Re-HDPE composites with different WF contents ((**a**) 50% WF/Re-HDPE, (**b**) 60% WF/Re-HDPE, and (**c**) 70% WF/Re-HDPE).

Studies have shown that the AE parameters such as amplitude, ringing counts, accumulative ringing counts, and accumulative energy can reflect the evolution damage and fracture process of the WF/Re-HDPE composite [4,28,29]. Figure 6 shows the AE amplitude, ringing counts, and stress-strain curves of the 50%, 60%, and 70% WF/Re-HDPE composites. It can be seen that there were no AE events at the initial stage of loading; however, they gradually appeared with increasing stress-strain showing lower amplitude. With the further increase of stress-strain, AE events gradually increased and tended to be stable. Thereafter, as the stress increased to the vicinity of the maximum value, AE events suddenly increased and the amplitude increased rapidly (the maximum value was about 100 dB). In addition, the AE events gradually decreased with an increasing WF content and the average amplitude significantly decreased; however, the maximum amplitude value was not significantly different. This was mainly caused by the enhanced fiber supporting effect on the matrix as the fiber content increased and the increased rigidity of WF/Re-HDPE composite. However, as the WF content increased, the agglomerated fiber results in a reduction in interfacial adhesion (as shown in Figure 7) [30]. The deformation and damage of the matrix was more serious, whereas the fiber breakage was decreased; hence, the amplitude and activity of AE decreased.

Figure 6d–f demonstrate that first, the AE ringing counts slowly increased with the increasing stress-strain, stabilized, and then suddenly increased (most did not exceed 100) as the stress reached the maximum to fracture. In addition, the ringing counts of the AE event was significantly reduced as the WF content increased. This is mainly because the matrix deformation and fiber debonding were the main damage modes during the damage process of WF/Re-HDPE composites. As the WF content increased, the dispersion of fibers in the matrix decreased and the mechanical interlocking of fiber-matrix reduced, which led to a reduction in the debonding of fiber-matrix during the damage process (as shown in Figure 7). Moreover, as the WF content increased, the ringing counts of the events with the ringing count above 100 had a tendency to decrease. This indicates that the signal impulse generated by the fracture of the WF/Re-HDPE composite decreased with the increasing WF content. This was primarily because the distance between the macromolecular chains of the matrix increases with the increasing WF content and the bonding strength decreased, leading to a decrease in the signal impulse and duration of AE event.

**Figure 8:**
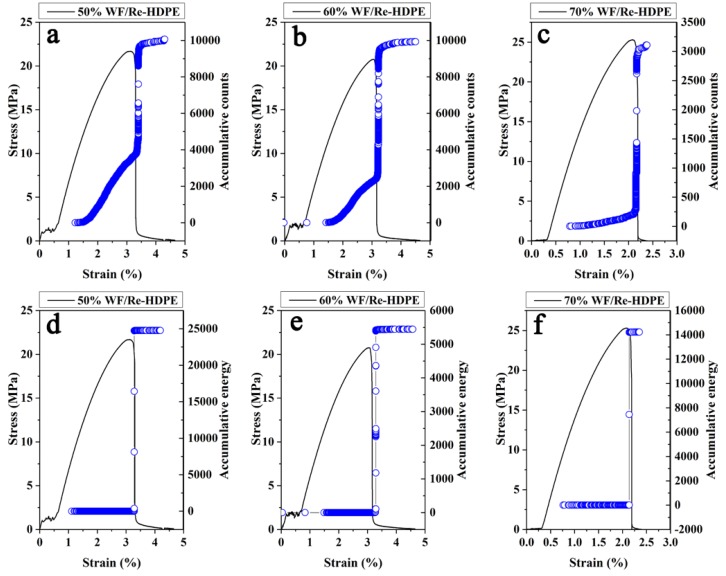
The AE accumulative ringing counts, accumulative energy, and stress-strain curve of WF/Re-HDPE composites with different WF content. The blue circle represents accumulative counts and accumulative energy in (**a**–**f**), respectively.

Figure 8 shows the AE accumulative ringing counts, accumulative energy, and stress-strain curves of the 50%, 60%, and 70% WF/Re-HDPE composites. As the stress-strain increased, the accumulative ringing counts changed in four stages: slow increase in the first stage, rapid increase in the second stage, sudden increase in the third stage (fracture stage), and gentle increase in the fourth stage (fracture later period). Furthermore, the accumulative ringing counts increased with increasing WF content but the increase rate in the second stage became flat, whereas the fiber-matrix debonding was the main damage mode of composites in this stage. These results further confirm that the fiber-matrix debonding decreases with increasing WF content during the damage process.

According to Figure 8d–f, the accumulative energy shows three major stages under the bending damage process. In the first stage, no energy was generated, as the stress-strain was low. The testing WF/Re-HDPE specimen only appeared to have undergone matrix deformation to generate potential energy. Then, the energy and damage slowly increased with the increasing stress-strain in the second stage and the energy of the AE signal suddenly increased in the third stage (fracture stage). This damage and fracture mode indicates that the WF/Re-HDPE composite is a brittle fracture material. Moreover, the total strain energy reduced during the damage and fracture process, however, the maximum stress was slightly increased. This is mainly because the rigidity of the composite increases as the WF content increased [30]. However, the debonding of fiber-matrix and the accumulative damage reduced with increasing WF content. Therefore, as the stress increased, the released strain energy reduced during the damage and fracture process.

### 3.3. AE Characteristics in Repeated Bending Loading Process

When the material is loaded and no clear AE event occurs until the repeated load reaches the original maximum load, this AE irreversible property is called as the Kaiser effect [31,32,33]. On the other hand, if a clear AE event occurs before the repeated load reaches the original maximum load, it is called as the Felicity effect [34]. Felicity ratio refers to the ratio of the repeated load (load that produces the AE signal in repeated loading process) to the original maximum load. Better is the damage resistance of the composites, larger is the Felicity ratio; this has become the basis for the evaluation of the damage degree of polymer composites [34]. A Felicity ratio greater than 1 indicates that the Kaiser effect is true, and a value less than 1 indicates that the Felicity effect is true. A smaller Felicity ratio indicates more severe damage or more structural defects of the composites. A Felicity ratio less than 0.95 is often used as an important criterion for excessive acoustic emission in some composites [35].

**Figure 9:**
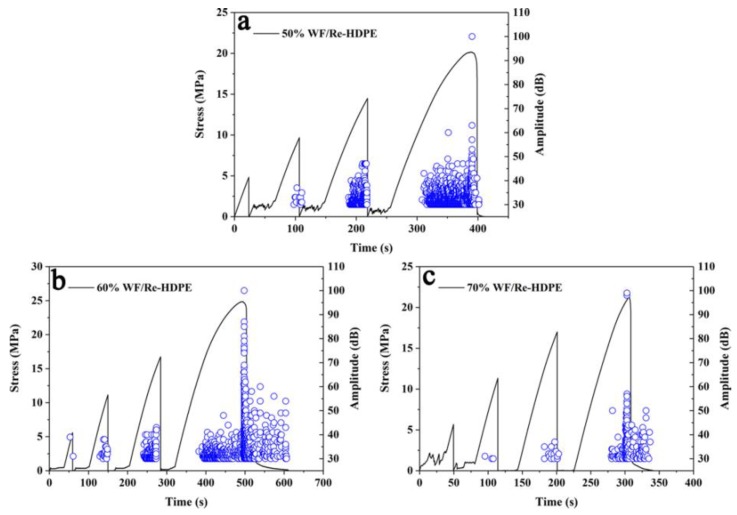
Time-strain curve and AE amplitude of WF/Re-HDPE composites with different WF content. The blue circle represents amplitude in Figure 9.

**Figure 10:**
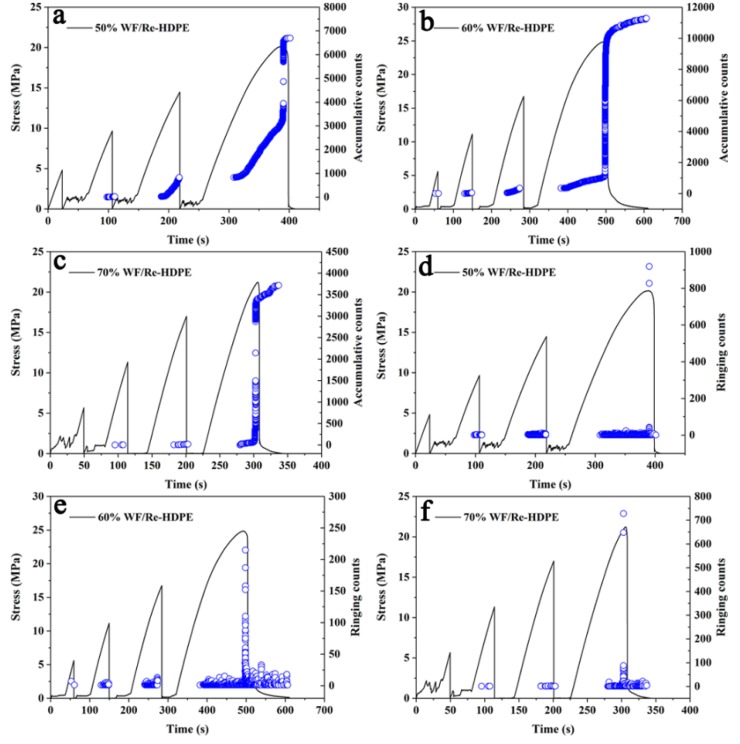
Time-strain curve, AE accumulative counts, and ringing counts of WF/Re-HDPE composites with different WF contents. The blue circle represents accumulative counts and ringing counts in (**a**–**f**), respectively.

The time variation of stress, AE amplitude, and accumulative ringing counts for 50%, 60%, and 70% WF/Re-HDPE composites is shown in Figure 9 and Figure 10. The Felicity ratios of the WF/Re-HDPE composites are presented in Table 2. The Felicity ratios of WF/Re-HDPE composites under the first repeated loading were gradually reduced with the increasing WF content, which indicates that the accumulative damage memory capacity of the composites had an increasing trend. Among these, the Felicity ratio of the 50% and 60% WF/Re-HDPE composite under the first repeated loading was greater than 1. These results indicate that these two levels of WF/Re-HDPE composites have a Kaiser effect in the initial stage of repeated loading. The damage was reversible for loads less than 25% of the maximum load; the composites had no memory for this damage. However, the Felicity ratio of the 70% WF/Re-HDPE composite under the first repeated loading was 0.93 (less than 0.95), which indicates that a load of less than 25% of the maximum load can cause irreversible damage to 70% WF/Re-HDPE composites. Furthermore, the composites had a strong memory capacity for this damage and the mechanical properties of the composites were greatly affected by the damage. The Felicity ratio of these three WF/Re-HDPE composite levels under the second repeated loading was less than 1, however, the Felicity ratio for 50% and 60% WF/Re-HDPE composites were 0.98 and 0.99 (greater than 0.95), respectively. This suggests that a load more than 50% of the maximum load could cause irreversible damage to the two levels of WF/Re-HDPE; however, the composites had a weaker memory for the damage. Then, the Felicity ratio of these three levels of WF/Re-HDPE composites gradually decreased with the increase in repeated loading times and stress, and the values were less than 0.95. The irreversible damage of the composite was gradually increased, the memory capacity for the damage was gradually enhanced, and the repeated load had an increasing influence on the mechanical properties of the composites.

Moreover, with the increase in repeated loading times and stresses, the AE events of the WF/Re-HDPE composites gradually decreased, while the amplitude and the ringing counts gradually increased, indicating that the damage degree of WF/Re-HDPE composites was increased. When the loading was less than 75% of the maximum load, there was no AE signal for 50% and 60% WF/Re-HDPE composites until the repeated loading stress reached the last maximum loading stress. Thereafter, the signals appeared after stress exceeded the last maximum loading stress. In addition, the position of the AE signal was the same for the last loading process at the initial stage, which further confirms the Kaiser effect and the Felicity effect of the WF/Re-HDPE composites. The 70% WF/Re-HDPE composite had an AE signal before the first repeated loading reached the last maximum strain, indicating that the composite had already produced a memory to the damage caused by the 25% loading and that the repeated loading had accumulated damage. In addition, Figure 10a–c also show a slope in the initial stage of accumulative ringing counts-time curve during the repeated loading process which is same as the slope in the last loading process, which further proves that the WF/Re-HDPE composites have a Felicity effect.

The above results indicate that both the Kaiser effect and the Felicity effect exist during the repeated loading of high-filled WF/Re-HDPE composites. The composites under lower repeated load mainly produced reversible damage at less WF content and had an irreversible damage at larger load or more repetitions, while the lower load could cause irreversible damage at high WF content. The 50% and 60% WF/Re-HDPE composites produced irreversible damage when the load exceeded 75% of the maximum load, while the 70% WF/Re-HDPE composites produced irreversible damage when the repeated load was less than 25% of the maximum load. However, these three levels of WF/Re-HDPE composites had memory for the damage caused by repeated loads. This may be because of the increase of WF content and the decrease of matrix, which resulted in the reduction of the fiber-matrix interface bonding strength. The fiber debonding occurred under a lower load and an irreversible damage was produced.

## 4. Conclusions

We combined the AE technique and SEM analysis to study the damage and fracture mode and mechanism of high-filled WF/Re-HDPE composites under a three-point bending test. The damage characteristics, damage location, and failure mechanism of the composites with different WF content were analyzed. The results showed that AE technology can assist in understanding the evolution of the damage and fracture process of WF/Re-HDPE composites and determine the damage degree, damage accumulation, and damage mode. The damage and fracture modes of high-filled WF/Re-HDPE composites under a three-point bending load were mainly matrix deformation, fiber breakage, interface delamination (fiber debonding), fiber pull-out, and matrix cracking. In addition, the damage and fracture process presents three main stages: the damage begins to occur in the first stage; less AE signal appears. Then, the damage accumulated in the second stage and the AE signal for different damage mode gradually increased. The third stage was the fracture stage, where the damage to the matrix and fiber was seriously exacerbated until the fracture as the AE signal showed a more pronounced change. Moreover, the Kaiser effect and the Felicity effect existed in the repeated loading of high-filled WF/Re-HDPE composites. The higher the WF content, the more severe the Felicity effect of the WF/Re-HDPE composites, the greater the accumulative damage caused by the repeated load, and the higher the influence on the mechanical properties of the composites. For WF/Re-HDPE composites with relatively low WF content, a low repetitive load mainly produced reversible damage, large load or more repetitions could cause irreversible damage, while the lower repeated loads could cause irreversible damage for WF/Re-HDPE composites with higher WF content.

## Figures and Tables

**Table 1:** Characteristics of AE signals corresponding to several damage modes.

Signal Type	Rise-Time/μs	Duration/μs	Ringing Counts	Absolute Energy·10^−9^ J	Amplitude/dB
Matrix deformation	8.95	48.63	5.34	76.46	34.31
Fiber breakage	2.13	108.47	13.70	339.31	55.29
Interface delamination	40.43	252.72	24.96	229.54	45.61
Interface friction	75.92	864.25	39.53	2577.12	48.36
Matrix cracking	577.84	2358.92	84.88	3479.46	61.38

**Table 2:** Felicity ratio of WF/Re-HDPE composites with different WF content.

Sample	Loading Times	Maximum Strain%	Strain at AE Signals Generated%	Felicity Ratio
50%WF/Re-HDPE	1	0.37	—	—
50%WF/Re-HDPE	2	1.30	1.16	3.15
50%WF/Re-HDPE	3	1.75	1.27	0.98
50%WF/Re-HDPE	4	2.67	1.41	0.80
60%WF/Re-HDPE	1	0.92	0.83	—
60%WF/Re-HDPE	2	1.41	1.09	1.18
60%WF/Re-HDPE	3	2.10	1.40	0.99
60%WF/Re-HDPE	4	3.28	1.53	0.73
70%WF/Re-HDPE	1	0.77	—	—
70%WF/Re-HDPE	2	1.01	0.72	0.93
70%WF/Re-HDPE	3	1.35	0.91	0.90
70%WF/Re-HDPE	4	1.64	1.08	0.80
